# Utility of the Neurobehavioral Cognitive Status Examination (COGNISTAT) in differentiating between depressive states in late-life depression and late-onset Alzheimer’s disease: a preliminary study

**DOI:** 10.1186/s12991-016-0091-5

**Published:** 2016-01-20

**Authors:** Yoshiaki Tsuruoka, Michio Takahashi, Masatoshi Suzuki, Koichi Sato, Yukihiko Shirayama

**Affiliations:** Department of Psychiatry, Teikyo University Chiba Medical Center, 3426-3 Anesaki, Ichihara, 299-0111 Japan; Shirayurikai Otaki Hospital, 786 Uehara, Otaki, 298-0223 Japan

**Keywords:** Alzheimer’s disease, COGNISTAT, Cognition, Late-life depression, MMSE, HAM-D

## Abstract

**Background:**

It is often difficult to differentiate between the depressive states seen in late-life depression and late-onset Alzheimer’ disease (AD) in the clinical setting.

**Methods:**

Thirty-four outpatients were recruited, all fulfilling the criteria of aged 65 years or above, scores of 14 or more on the Hamilton depression rating scale (HAM-D), and 26 or less on the Mini-Mental State Examination (MMSE). At the initial visit, they were administered the Neurobehavioral Cognitive Status Examination (COGNISTAT). At 1 month, a diagnosis of either senile depression (*n* = 24) or Alzheimer’ disease (*n* = 10) was made.

**Results:**

The COGNISTAT revealed that the late-life depression group showed significantly higher scores in orientation and comprehension subtests compared with the AD group. At the study endpoint (6 months after treatment), MMSE detected significant improvements in the late-life depression group (*n* = 15), but no changes in the late-onset AD group (*n* = 7). Scores for memory, similarities, and judgment on the second COGNISTAT were significantly improved in the depressed group, whereas calculation scores deteriorated significantly in the AD group.

**Conclusion:**

The COGNISTAT could prove useful in differentiating late-life depression from late-onset AD, despite similar scores on MMSE.

## Background

It is important to make a clinical distinction between late-life depression and Alzheimer’s disease (AD) in geriatric patients, because although both diseases exhibit dementia or cognitive dysfunction completed suicides show close association with major depressive illness, especially in the elderly [1]. Previous reviews have documented the typical clinical and cognitive presentation of late-life depression and AD [2, 3]. In primary care, the Mini-Mental State Examination (MMSE) is a frequently used and convenient assessment tool for global cognitive function [4]. The clock drawing test is also well used for assessing visuospatial disabilities in AD. For a more accurate assessment of dementia, it is necessary to utilize complex assessment tools, including Wechsler Adult Intelligence Scale (WAIS), Wechsler Memory Scale (WMS), and the Alzheimer’s Disease Assessment Scale (ADAS) [5].

The Neurobehavioral Cognitive Status Examination, COGNISTAT, is a short cognitive test for evaluating cognitive impairment in AD. Patients with AD showed significantly lower scores on many subtests of the COGNISTAT compared with healthy, elderly individuals [6, 7]. Furthermore, the total number of impaired scores on the COGNISTAT was useful for discriminating AD from non-AD dementia [8].

Depression in late-life is one of the most common mental disorders in old age. The prevalence rate of senile depression is almost similar to that of AD in elderly people in their seventh decade. Depressive symptoms were found in 27 % of patients with AD [9]. It has been shown that depressive symptoms are associated with cognitive impairment including information processing speed, executive function, episodic memory, visuospatial function, and language processing in elderly patients [2, 10–12]. Late-life onset depression is associated with a variety of cognitive impairments [2]. Late-life depression with cognitive impairment and the frequency and severity of depressive episodes increase the risk of dementia [13, 14]. Cognitive impairment in elderly depressive patients improves significantly after treatment with antidepressant drugs [15–17]. On the other hand, antidepressant therapy in AD patients showed no significant cognitive changes compared to placebo [18], but significantly improved depressive symptoms [19]. From a clinical point of view, it is likely that accurate discrimination between depression and AD could result in more appropriate and therefore more beneficial therapies for elderly patients [20–22].

Many studies have struggled to develop criteria for differentiating between dementia and depression, using various cognitive and neuropsychological tests [23–26]. However, few analyses have addressed the distinction between late-life depression and dementia in elderly depressed patients with cognitive dysfunction [23, 24]. Furthermore, in these studies, the two cohorts did not display similar clinical severity using MMSE. In this study, we investigated the utility of COGNISTAT for discriminating between late-life depression and late-onset AD using patients with similar scores on MMSE.

## Methods

### Subjects

All patients were recruited from the outpatient clinics of Teikyo University Chiba Medical Center (Ichihara, Chiba, Japan), and met the DSM-IV criteria (American Psychiatric Association) for a major depressive episode or AD. Inclusion criteria for this study were (1) a 26 and below score on the MMSE, (2) a 14 and above score on the 17-item Hamilton Rating Scale for Depression (HAM-D), and (3) aged above 65 years. In order to accurately determine the diagnosis, imaging data from CT, MRI, MRA, and/or SPECT were obtained in some cases. Patients with cerebral vascular disease were excluded from the study. We also tried to exclude patients with mild cognitive impairment [27]. This study was approved by ethics committee of Teikyo University Chiba Medical Center (study number 13–226), and performed in accordance with the declaration of Helsinki. All patients and their caregivers provided written informed consent.

### Study design

At the initial visit, 34 subjects were administered the COGNISTAT. Then, all subjects were diagnosed and divided into a late-life depression group (*n* = 24) or late-onset AD group (*n* = 10) during the first month of study. Diagnoses were not changed during this period. Next, patients were prescribed either antidepressant drugs or cholinesterase inhibitors.

After remission or 6 months of treatment (the study endpoint), a second COGNISTAT was administered to the late-life depression group (*n* = 15) and late-onset AD group (*n* = 7) to determine any changes in cognitive function. The late-life depression group comprised patients who received mirtazapine (*n* = 10), escitalopram (*n* = 3), and paroxetine (*n* = 2). The late-onset AD group received donepezil (*n* = 4), galantamine (*n* = 2), and paroxetine (*n* = 1).

For further analysis, we divided patients with late-life depression into remission group (*n* = 10) and non-remission group (*n* = 5), depending on whether they achieved remission during the study. We defined remission as a score of 7 or less on the HAM-D.

### Measurement of cognitive function

COGNISTAT is typically utilized for assessing multiple cognitive functions, using a screen-metric method [28]. Generally, it takes 15–20 min to administer. The COGNISTAT consists of 10 subsets, including orientation, attention, comprehension, repetition, naming, construction, memory, calculation, similarities, and judgment. The three components, namely comprehension, repetition, and naming, determine the language ability. The two components of similarities and judgment determine the ability to reason. However, these subtests lack the strength of detection compared with other neurocognitive tests, such as WMS and WAIS. The normative data of elderly people were developed to aid diagnosis of cognitive impairment, since aging influences some components of construction, memory, and similarities on the COGNISTAT [29–31].

### Statistical analysis

Multiple analysis of variance (MANOVA) was used to determine the simultaneous existence of significant differences. Statistical analysis was performed using Student’s *t*-test or paired t-test for parametric data, or the Chi square test for non-parametric data. Correlations among scores from COGNISTAT were examined using the Pearson correlation coefficient. Differences were considered significant when *p* values were less than 0.05 for ANOVA, *t*-test, and Chi square test, and 0.01 for correlation coefficient.

We calculated the sensitivity and specificity of COGNISTAT for late-life depression, using COGNISTAT subtests with a significant difference between late-life depression and AD in a cross-sectional design. The sensitivity and specificity were defined as stated previously [32]. The criteria of normal or impaired states on the COGNISTAT were defined using healthy elderly individuals as standards [33].

Sensitivity to late-life depression was defined as the following: the results of COGNISTAT suggest late-life depression when the patient was diagnosed as having depression. Sensitivity was calculated by the ratio of patients with late-life depression with scores above the set criteria to the total number of patients with late-life depression.

Specificity to late-life depression was defined as the following: the results of COGNISTAT did not suggest late-life depression, when the patient was diagnosed as having AD. Specificity to late-life depression was calculated by the ratio of AD patients with scores below the set criteria to the total number of AD patients.

Criteria for calculating sensitivity and specificity are an orientation score of 10 and/or a comprehension score of 5, because of the significant differences on both of scores between late-life patients and late-onset AD patients.

## Results

### Neuropsychological test results at baseline

**Fig. 1:**
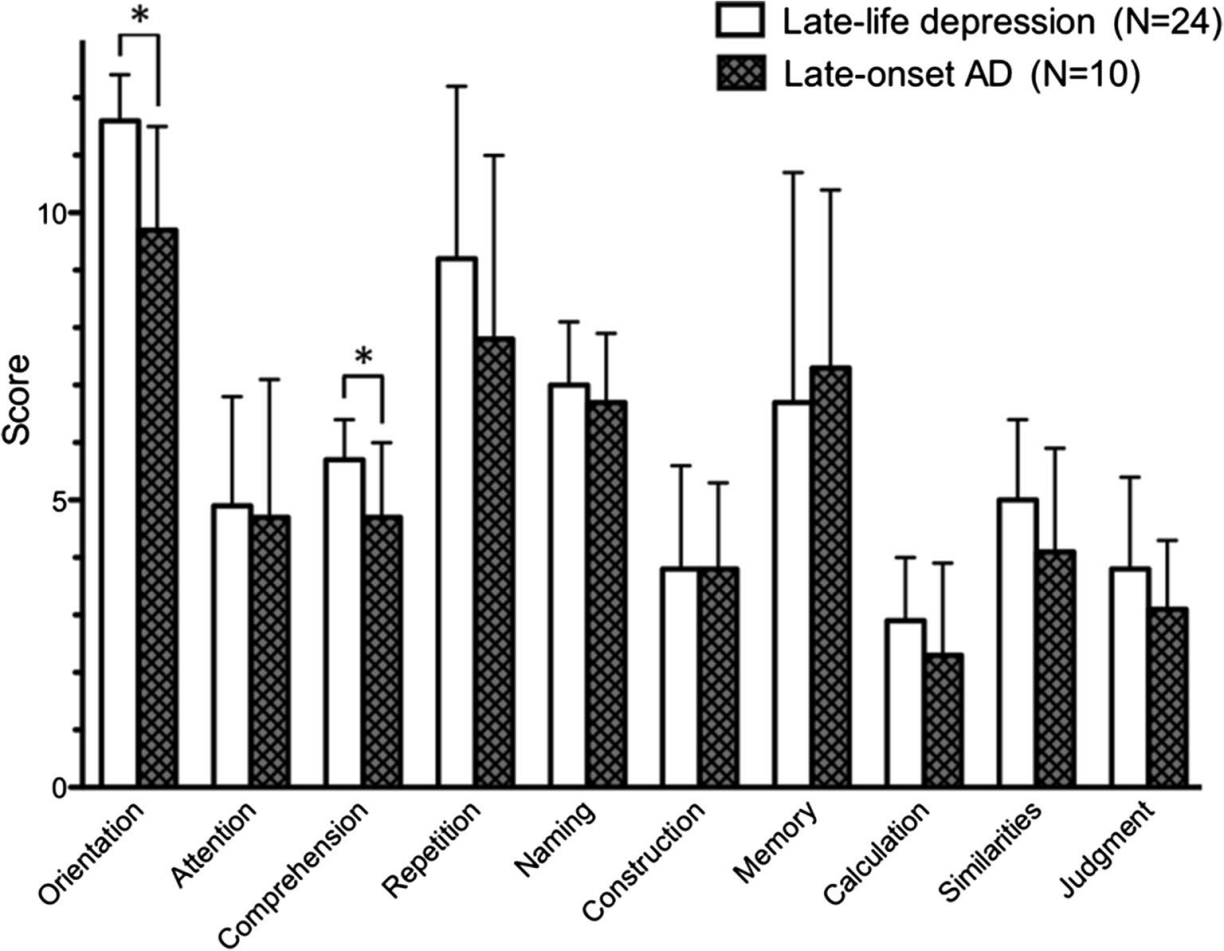
The COGNISTAT scores at baseline. Student’s t-test showed significant differences between the late-life depression group and late-onset Alzheimer’s disease group for orientation and comprehension subtests. * *p* < 0.05

Of the 34 patients, 24 were diagnosed as suffering major depressive disorder, and 10 were diagnosed as having AD, 1 month after their first study. The baseline demographic characteristics are presented in Table 1. The patients with late-life depression and late-onset AD did not differ in age, sex, or MMSE scores. However, late-life depression patients showed significantly higher HAM-D scores compared with late-onset AD patients. On the COGNISTAT components, MANOVA indicated a significant group effect (*F* = 3.532, *p* = 0.006). Subsequent Student’s *t*-test demonstrated that the late-life depression group had significantly higher scores for orientation (*t* = 3.141, *p* = 0.010) and comprehension (*t* = 2.262, *p* = 0.045) on the COGNISTAT (Fig. 1).

**Table 1:** Comparison of characteristics between the late-life depression group and Alzheimer’s disease group

	Late-life depression group (*n* = 24)	Late-onset AD group (*n* = 10)	*P*
Sex (M/F)	6/18	5/5	0.156
Age (years)	72.4 ± 7.1	76.0 ± 5.9	0.171
HAM-D	21.3 ± 7.1	16.1 ± 4.7*	0.043
MMSE	23.4 ± 2.8	21.9 ± 2.8	0.163

Data are shown as mean ± SD

*HAM-D* the 17-item Hamilton Rating Scale for Depression, *MMSE* Mini-Mental State Examination, *AD* Alzheimer’s disease

* *p* <0.05 by Student’s *t*-test

For all participants, MMSE scores displayed significant correlation with orientation and calculation scores on the COGNISTAT (Table 2), whereas HAM-D showed no significant correlation with MMSE or COGNISTAT subcomponents (data not shown).

**Table 2:** Correlation between MMSE and COGNISTAT subtests in all patients (*n* = 34)

	1	2	3	4	5	6	7	8	9	10	11
	MMSE	Ori	Atten	Comp	Rep	Nam	Const	Mem	Cal	Sim	Judg
				Language						Reasoning	
1. MMSE		0.542*	0.247	0.337	0.338	0.275	0.166	0.409	0.590*	0.283	0.227
2. Orientation			0.253	0.341	0.303	0.419	0.003	0.191	0.395	0.187	0.309
3. Attention				0.194	0.460*	0.416	0.442*	0.099	0.255	0.359	0.529*
4. Comprehension					0.363	0.099	0.075	0.164	0.502*	0.546*	0.497*
5. Repetition						0.374	0.326	0.008	0.369	0.318	0.32
6. Naming							0.393	0.082	0.275	0.437*	0.368
7. Construction								0.09	0.316	0.29	0.514*
8. Memory									0.037	0.172	0.077
9. Calculation										0.603*	0.614*
10. Similarities											0.599*
11. Judgment											

Data are shown as mean ± SD

* *p* <0.01

*Ori* orientation subtest, *Atten* attention subtest, *Comp* comprehension subtest, *Rep* repetition subtest, *Nam* naming subtest, *Const* construction subtest, *Mem* memory subtest, *Cal* calculation subtest, *Sim* similarities subtest, *Judg* judgment subtest

Furthermore, we calculated sensitivity and specificity to assess the utility of this method for differentiating between late-life depression and late-onset AD, using scores from orientation and/or comprehension subtests. When the standard for late-life depression was defined as a score of 10 or more for orientation, sensitivity was 96 % and specificity was 50 %. When the standard criterion of late-life depression was defined as 5 or more for scores on the comprehension, sensitivity was 96 % and specificity was 40 %. Moreover, when the standard for late-life depression was set at scores of 10 or more on orientation, and 5 or more on comprehension, sensitivity was 92 % and specificity was 60 %.

In the MMSE, when a cut-off point sets the standard criterion for late-life depression at a score of 24 or more, sensitivity and specificity of MMSE to late-life depression were 58 % and 60 %, respectively. When a cut-off point sets the standard criterion at a score of 22 or more, sensitivity and specificity of MMSE to late-life depression were 79 % and 50 %, respectively.

### Comparison of cognitive function between baseline and endpoint in each group

Fifteen of 24 patients (62.5 %) with late-life depression and seven of 10 patients (70.0 %) with late-onset AD completed this longitudinal research. At the study endpoint, defined as 6 months after treatment, MMSE detected significant improvements in the senile depression group (from 22.7 to 24.9, *t* = 2.402, *p* = 0.031), whereas no changes were observed in the late-onset AD group (from 22.1 to 21.3, *t* = 0.446, *p* = 0.671). HAM-D demonstrated significant improvement in the late-life depression group (from 21.6 to 9.3, *t* = 10.931, *p* <0.001), and a non-significant trend for improvement in the AD group (from 16.6 to 10.9, *t* = 1.498, *p* = 0.185).

**Fig. 2:**
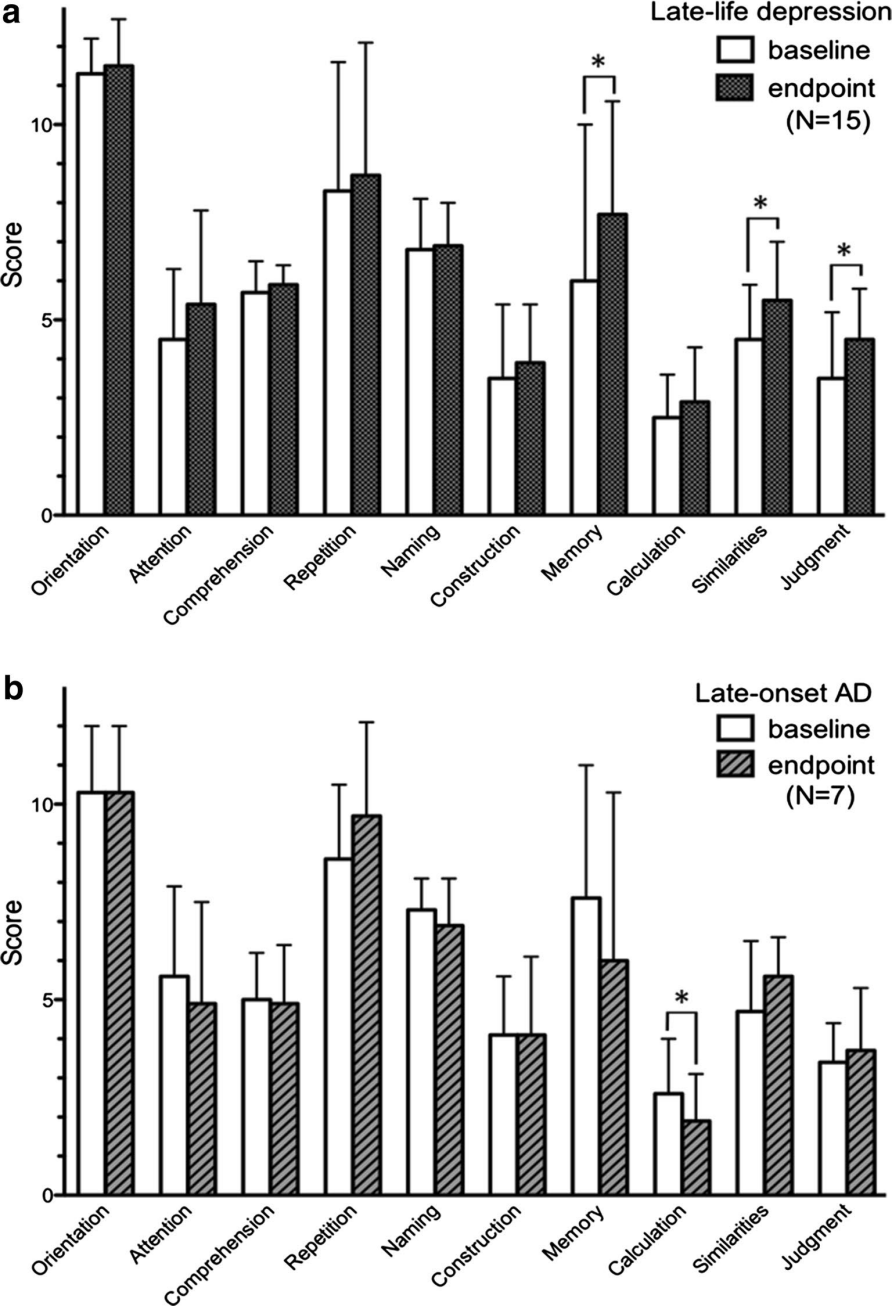
The COGNISTAT scores at baseline and endpoint.** a** Comparison of COGNISTAT scores at baseline and endpoint in the late-life depression group. Scores for memory, similarities, and judgment were significantly higher at endpoint compared with baseline.** b** Comparison of COGNISTAT scores at baseline and endpoint in the late-onset Alzheimer’s disease group. Score for calculations was significantly lower at endpoint than at baseline. * *p* < 0.05

As shown in Fig. 2, between baseline and endpoint, the late-life depression group showed significant improvement of some cognitive functions, such as memory (*t* = 2.385, *p* = 0.032), similarities (*t* = 2.739, *p* = 0.016), and judgment (*t* = 2.739, *p* = 0.016). The late-onset AD group showed a significant reduction in calculation scores (*t* = 2.500, *p* = 0.047) at endpoint, compared to baseline.

### Effects of cognitive performance at baseline on the treatment outcome in late-life depression

Of the 15 patients with late-life depression who completed the 6-month study period, 10 were classified into a remitted group and 5 into a non-remitted group. The remitted group, who scored 7 and below on the HAM-D, showed recovery on the MMSE (*t* = 2.493, *p* = 0.034) and judgment (*t* = 3.284, *p* = 0.009), while the non-remitted group, who scored 10 and above on the HAM-D, showed a similar trend without statistical significance in these two categories.

## Discussion

The first finding of this study was that late-onset AD patients suffered worse impairment of orientation and comprehension as measured on the COGNISTAT, relative to patients with late-life depression, despite no significant difference in MMSE scores at baseline between two groups. The MMSE score showed significant correlation with orientation and calculation scores, but not the comprehension score, indicating that orientation and calculation subtests share stronger relationships with dementia symptoms in this test. Interestingly, the calculation subtest showed a strong relationship with subtests for comprehension, similarities, and judgment on the COGNISTAT. However, HAM-D scores showed no significant relationship with any scores on the COGNISTAT in either patient group (data not shown). It appears therefore that subtests for orientation and comprehension on the COGNISTAT are capable of discriminating late-life depression from late-onset AD, independent of depressive symptoms, in patients suffering both a depressive state and cognitive impairment. A previous study reported that subtests for comprehension, construction, and memory on the COGNISTAT were useful for differentiating depression from dementia [23], a finding which is in partial agreement with this study. It should be noted that this study recruited patients in early onset of depression and cognitive dysfunction. Thus, the disease severity of the study subjects was light to moderate, potentially increasing the difficulty of discriminating between the two types of disease. Further studies will be needed to clarify this issue.

The sensitivity and specificity of COGNISTAT to late-life depression were 92 % and 60 %, respectively, when the standard scores for late-life depression were set at 10 or more on orientation, and 5 or more on comprehension. These findings indicate that COGNISTAT is able to discriminate late-life depression from late-onset AD in elderly patients. Sensitivity and specificity of MMSE to late-life depression were 79 % and 50 %, respectively, when the standard scores for late-life depression were set at a score of 22 or more. Previous studies showed that COGNISTAT was superior to MMSE in determining sensitivity to cognitive impairment [34, 35], but inferior to MMSE in specificity [35], which is partially in agreement with the present study.

The second finding was that the late-life depression group exhibited significant improvement in memory, similarities, and judgment scores on the COGNISTAT, whereas the late-onset AD group exhibited significant worsening in the calculation subtest at the endpoint. It is plausible that the reduction in subtests for memory, similarities, and judgment, was due to cognitive dysfunction in the depressive state. As shown in Table 2, similarities and judgment scores were strongly related to the comprehension subtest, which is one of two important factors for discriminating late-life depression from late-onset AD at baseline. This is supported by previous studies which found that antidepressant treatment improved global cognitive function in elderly depressed patients [16, 17, 33].

The late-life depression group showed significant recovery in HAM-D and to a lesser extent in MMSE scores. This is in good agreement with previous studies showing that cognitive dysfunction persists to some extent, even though depressive symptoms show sufficient improvement in geriatric depression [15, 36]. However, the late-onset AD group showed non-significant improvement of depressive symptoms without any changes in MMSE scores. In a clinical context, the pattern of changes is very similar between the two groups. Therefore, neither HAM-D nor MMSE is sufficient for discrimination between late-life depression and late-onset AD. In a previous study, the memory scale and at least one other scale on the COGNISTAT were useful for identifying dementia, including AD [8]. However, prior to treatment, depressed patients also showed reduced memory performance [17]. Cued recall was useful in differentiating early AD and cognitive dysfunction from depression in elderly individuals [37]. A decline in performance on the test of episodic memory and subsequent declines in tests for executive function were observed before diagnosis in preclinical AD [38]. In this study, baseline memory scores on the COGNISTAT were not impaired in late-onset AD patients with reference to the criteria for elderly individuals. This might well be due to cognitive dysfunction within the present cohort.

Both similarities and judgment subtests on COGNISTAT are characterized by reasoning. Previous studies have shown that these subtests reflect executive function, working memory, planning, inhibition, and concept generation [39–41]. Although acetylcholinesterase inhibitor therapy failed to improve executive function in AD patients [42], antidepressant therapy improved cognitive impairment in elderly patients with major depression [43]. Therefore, it is likely that in the late-life depression group, impairment of similarities and judgment on the COGNISTAT could be due to a diminished reasoning.

Neuropsychological dysfunction of late-life depression is typified by slowed information processing, which influences all domains of cognition, including memory and executive function [10]. Furthermore, slow cognitive processing speed is the core cognitive deficit in late-life depression and is closely followed by executive function [11]. Therefore, improvement of memory, similarities, and judgment scores from late-life depressed patients in this study may be the result of improved information processing speed, mediated by antidepressant therapy. Further studies will be needed to examine the relationship between cognitive function on the COGNISTAT and information processing speed, as it was not addressed in this study.

This study has several limitations. First, the sample size is relatively small. Second, there was a significant difference on the HAM-D score between late-life depression and AD groups at baseline. This may reflect the fact that AD patients tend to underestimate their depressive symptoms [44], and the depressive state in AD patients is typically less severe and becomes less pronounced as the dementia symptoms progress [3]. Finally, patients in the AD group do not suffer mild cognitive impairment by clinical diagnosis, whereas the depression group may contain patients with mild cognitive impairment, since it was reported that patients with mild cognitive impairment suffer from major depression (20 %) [45].

## Conclusion

COGNISTAT is capable of differentiating late-life depression from late-onset AD, based on higher scores in orientation and comprehension subtests, among patients with both depressive symptoms and cognitive dysfunction at baseline, despite similar scores on MMSE. At endpoint, patients with late-life depression showed significant improvement in subtests for memory, similarities, and judgment, whereas patients with late-onset AD showed significant worsening in the calculation subtest compared to baseline.
